# Paramagnetic properties of carbon-doped titanium dioxide

**DOI:** 10.1186/1556-276X-7-333

**Published:** 2012-06-21

**Authors:** Anton A Minnekhanov, Daria M Deygen, Elizaveta A Konstantinova, Alexander S Vorontsov, Pavel K Kashkarov

**Affiliations:** 1 Physics Department, M. V. Lomonosov Moscow State University, Leninskie Gory, Moscow, 119991, Russia; 2 Russian Research Center Kurchatov Institute, 1, Akademika Kurchatova pl, Moscow, 123182, Russia

**Keywords:** carbon-doped titanium dioxide, electron paramagnetic resonance, defect

## Abstract

This paper reports the experimental results on paramagnetic properties of carbon-doped titanium dioxide. The electron paramagnetic resonance study of the samples has been carried out both in dark and under illumination. The nature of defects and their dynamics under illumination of carbon-doped TiO_2_ samples is discussed.

## Background

Titanium dioxide, because of its non-toxicity and high catalytic activity in various photo-oxidation reactions, represents the most important semiconductor photocatalyst. However, its large band gap (approximately 3.2 eV) requires the use of UV light and, therefore, does not allow utilizing also the much larger visible part of solar light [1]. For this reason, during the last years, many attempts were made to obtain a modified titania which is photocatalytically active also with visible light. Typical examples are surface modification by transition metal ions [2,3] and nonmetallic elements such as carbon [4], nitrogen [5], and sulfur [6]. All these novel materials photocatalyze complete visible light mineralization of various pollutants in water and air, and some nitrogen- or carbon-doped titania powder are active even in diffuse indoor daylight of very weak light intensity [4,5]. Experimental and theoretical results indicated that these dopants generate localized energy levels (surface states) just above the valence band from which visible light excitation becomes feasible [4,5]. Due to these intra-bandgap states, the carbon-doped titania exhibits a weak sub-bandgap light absorption starting already at about 700 nm. These materials contained 0.4% to 4.0% carbon in the form of carbonate and elemental carbon as indicated by X-ray photoelectron spectra [4]. To characterize these C-doped materials in more detail and to obtain basic information on the nature of the carbon dopant, we investigated the electronic properties by electron paramagnetic resonance **(**EPR) spectroscopy. This very sensitive method allows detection and characterization of paramagnetic defects which may be of significant importance for the photocatalytic properties [7-12]. For example, Li et al. ascribed the visible light activity of C-doped TiO_2_ to the presence of oxygen vacancies as suggested by the EPR detection of Ti^3+^ species [8]. However, it is noticed that the calculation of *g*-values is incorrectly performed, and details on the wavelength of exciting light is missing. In the following, we compare EPR data for a series of C-doped titania powders to clarify the nature of the paramagnetic centers and their change upon illumination under well-defined conditions.

## Methods

Bulk-modified material, C-TiO_2_-1, containing 0.42 wt.% carbon was prepared through hydrolysis of titanium tetrachloride with tetrabutylammonium hydroxide followed by calcination at 400°C for 1 h and at 350°C for 2 h, respectively [4]. The surface-modified sample C-TiO_2_-2 containing 1.05 wt.% carbon was prepared by suspending 3 g of titanium dioxide (Kerr-McGee Pigments GmbH, Krefeld, Germany) and 4 ml of glycerol in 50 ml of distilled water. After sonicating for 30 min, the suspension was stirred magnetically overnight, and the solvent was removed. Thereafter, the residue was crushed to a fine powder and calcined in air for 30 min at 300°C. The sample C-TiO_2_-3 was a commercially available surface-modified material and contained 0.46 wt.% of carbon (Kronos Incorporated, Asse-Zellik, Belgium). All weight percentages of carbon reported in this paper were obtained by elemental analysis. According to X-ray diffraction, all samples consist of the anatase modification [4].

EPR spectra were detected by the standard Bruker EPR spectrometer ELEXSYS-500 (X-band, sensitivity is around 10^10^ spin/G; Bruker BioSpin, Moscow, Russia). Mn^2+^ in MgO was employed as reference for *g*-values. After filling the powder into a quartz tube, air was pumped off at 5⋅10^−6^ Torr during 30 min followed by filling with He gas up to a pressure of 10^−1^-10^−2^ Torr and sealing of the tubes. The samples were investigated at 300 and 5 K.

The samples were illuminated (*in situ*) at 5 K with a 100-W tungsten halogen lamp in the spectral range of 400 to 1,000 nm.

## Results and discussion

**Figure 1:**
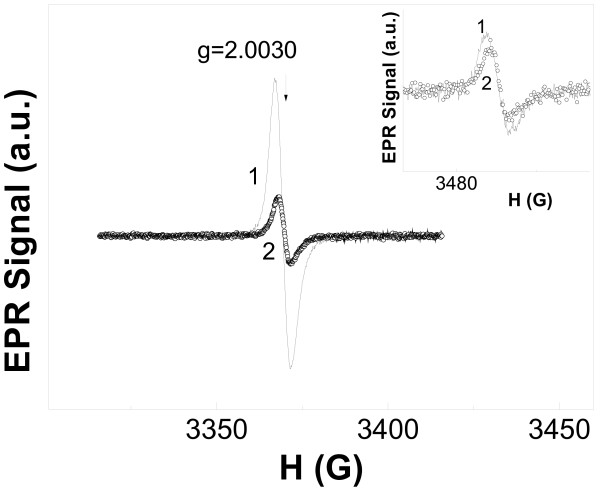
**EPR spectra of surface-doped samples at 5 K**. 1, C-TiO_2_-2; 2, C-TiO_2_-3. Inset shows the same samples but at 300 K. Arrow shows the position of *g*-values.

EPR spectra of all samples did not change during months being conserved at room temperature in darkness. EPR signals of surface carbon-doped TiO_2_ samples (C-TiO_2_-2, C-TiO_2_-3) are practically isotropic and are characterized by rather high intensity. Their parameters are equal to the following: *g* = 2.0030 ± 0.0005; the line width Δ*H*_2_(C-TiO_2_-2) = 4.7 ± 0.2 G and Δ*H*_3_(C-TiO_2_-3) = 3.7 ± 0.2 G (Figure 1). Samples with higher carbon concentration (C-TiO_2_-2) have higher content of paramagnetic centers: N_2_(C-TiO_2_-2) = 2·10^16^ spin/g, N_3_(C-TiO_2_-3) = 4·10^15^ spin/g. Similar EPR signals were reported in [13-15] for the carbon dangling bonds in amorphous carbon particles. Another possible explanation of the nature of the such-type EPR signal can be found in [8,16]. The authors of [8,16] ascribed a symmetric single line with *g* = 2.0030 to the conduction electrons trapped by oxygen vacancies. Unfortunately, a mechanism of such process is not clear from both papers.

**Figure 2:**
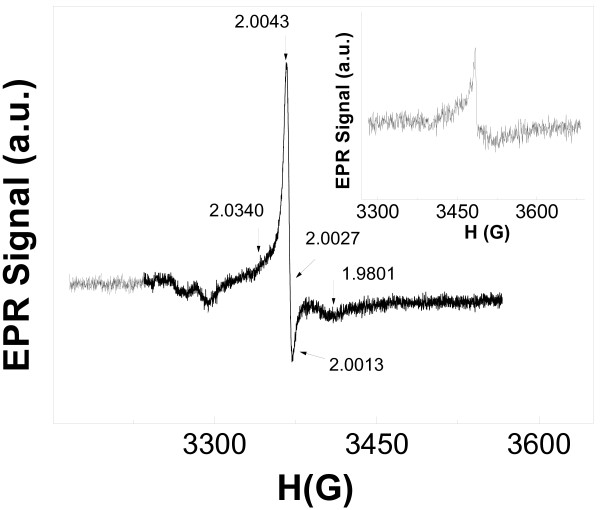
**EPR spectrum of volume-doped samples C-TiO**_**2**_**-1 at 5 K**. Inset shows the same sample but at 300 K. Arrows show the position of *g*-values.

It should be mentioned that the shape of EPR spectrum and the main parameters were unchanged for both samples at different temperatures: 300 and 5 K (Figure 1, inset). This fact reflects the negligible role of spin–lattice relaxation in these samples. The volume-doped samples (C-TiO_2_-1) had completely different EPR signals (Figure 2). The asymmetric shape of the signal is known for the 17e^−^ three atomic π-radical with *g*-factor values: *g*_1_ = 2.0042 ± 0.0005, *g*_2_ = 2.0027 ± 0.0005, and *g*_3_ = 1.9801 ± 0.0005. This signal can be assigned to CO_2_^−^ radicals, which were previously detected in MgO, NaHCO_2_, and KHCO_2_[17-19]. Seems, this anion-radical has been observed in C-TiO_2_ samples firstly. The EPR signal of CO_2_^−^ radicals was also detected at room temperature but with lower intensity (Figure 2, inset). We assume that CO_2_^−^ radicals are located in the interstitial sites of TiO_2_ lattice. Taking into account a shoulder of the EPR signal in a magnetic field within *g* = 2.0043-2.034 (Figure 2) and the absence of EPR signals from Ti^3+^ centers, one can propose the following mechanisms of CO_2_^−^ formation at the stage of C-TiO_2_-1 synthesis: CO_2_ + O^2−^(lattice) = CO_2_^−^ + O^−^(lattice); CO_2_ + Ti^3+^(lattice) = CO_2_^−^ + Ti^4+^(lattice). The *g*-values of O^−^ radicals are the following for various matrixes: *g*_1_ = 2.020-2.028, *g*_2_ = 2.009-2.019, and *g*_3_ = 2.002-2.0073 [11,12,20,21]. Therefore, we assume that the shoulder of the EPR line mentioned above can be assigned to EPR signal of O^−^ radicals. The content of paramagnetic centers in C-TiO_2_-1 samples was equal to N_1_(C-TiO_2_-1) = 10^15^ spin/g.

**Figure 3:**
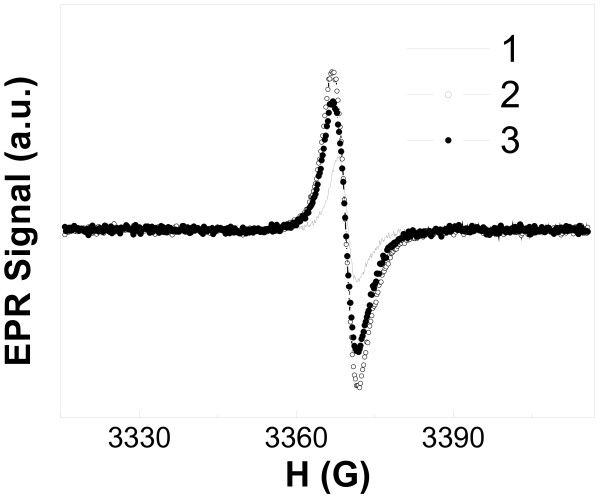
**EPR spectra of surface-doped sample C-TiO**_**2**_**-2 at 5 K**. 1, before illumination; 2, under illumination; 3, after illumination.

Under illumination of all doped-TiO_2_ samples, a growth of EPR signal intensity was registered. As an example, the effect of illumination of C-TiO_2_-2 sample on the EPR spectrum is shown in Figure 3. Partial reduction of the EPR signal intensity has been observed after illumination (Figure 3). Such changes of the EPR signal intensity under and after illumination can be explained due to a light absorbance by negatively or positively charged carbon dangling bonds, which are located inside the energy gap of TiO_2_. During illumination, the dangling bonds are changing to a neutral paramagnetic state; therefore, the spin density of paramagnetic centers increases. After illumination, density of paramagnetic centers decreases due to capture of electrons and holes by neutral paramagnetic centers.

## Conclusions

In summary, we should like to conclude that carbon-doped TiO_2_ samples have CO_2_^−^ radicals (C-TiO_2_-1) as well as with carbon defects (dangling bonds) on its surface (C-TiO_2_-2, C-TiO_2_-3). Additional energy levels of both interstitial carbon atoms or surface defects (carbon particles) should be located in the band gap of TiO_2_, as it was shown for titania doped with tiny metal nanoparticles of Cu, Pd, Pt and Ag [22-24], when doping of TiO_2_ led to formation of electronic surface states in semiconductor band gap. Such additional levels created by dopants can absorb visible light, increasing photosensitivity of the carbon-doped TiO_2_.

## Competing interests

The authors declare that they have no competing interests.

## Authors' contributions

AAM carried out the measurement of EPR spectra at 300 K, performed the analysis of these spectra and edited the final version of the manuscript. DMD participated in the measurement of EPR spectra at 5 K and performed the analysis of these spectra. EAK participated in the design of the study, performed the analysis and drafted the manuscript. ASV provided assistance of measurements under illumination *in situ*. PKK participated in the discussion of the results and provided financial support. All authors read and approved the final manuscript.

## Authors' information

EAK and PKK are both professors and doctorate degree holders in the Chair of General Physics and Molecular Electronics, Department of Physics, Lomonosov Moscow State University. ASV is a research associate. DMD is a PhD student, and AAM is a student at the same university.
